# Assessing the construct validity and responsiveness of Preference-Based Measures (PBMs) in cataract surgery patients

**DOI:** 10.1007/s11136-020-02443-3

**Published:** 2020-02-20

**Authors:** Katie Breheny, William Hollingworth, Rebecca Kandiyali, Padraig Dixon, Abi Loose, Pippa Craggs, Mariusz Grzeda, John Sparrow

**Affiliations:** 1 Population Health Sciences, Bristol Medical School, University of Bristol, Bristol, UK; 2 Department of Ophthalmology, Bristol Eye Hospital, Bristol, UK

**Keywords:** Cataract, EQ-5D, ICECAP-O, Bolt-on, Responsiveness, Validity

## Abstract

**Purpose:**

The validity and responsiveness of the EQ-5D-3L in visual conditions has been questioned, inspiring development of a vision ‘bolt-on’ domain (EQ-5D-3L + VIS). Developments in preference-based measures (PBM) also includes the EQ-5D-5L and the ICECAP-O capability wellbeing measure. This study aimed to examine the construct validity and responsiveness of the EQ-5D-3L, EQ-5D-5L, EQ-5D-3L + VIS and ICECAP-O in cataract surgery patients for the first time, to inform choice of PBM for economic evaluation in this population.

**Methods:**

The analyses used data from the UK Predict-CAT cataract surgery cohort study. PBMs and the Cat-PROM5 [a validated measure of cataract quality of life (QOL)] were completed before surgery and 4–8 weeks after. Construct validity was assessed using correlations and known-group differences evaluated using regression. Responsiveness was evaluated using effect sizes and analysis of variance to compare change scores between groups, defined by patient-reported and clinical outcomes.

**Results:**

The sample comprised 1315 patients at baseline. No PBMs were associated with visual acuity and only the ICECAP-O (Spearman’s rs =  − 0.35), EQ-5D-3L + VIS (rs =  − 0.42) and EQ-5D-5L (Value Set for England rs =  − 0.31) correlated at least moderately with the Cat-PROM5. Effect sizes of change were consistently largest for the EQ-5D-3L + VIS (range 0.34–0.41), followed by the ICECAP-O (range 0.20–0.34). Results indicated no improvement in responsiveness using the EQ-5D-5L (range 0.13–0.16) compared to the EQ-5D-3L (range 0.17–0.20).

**Conclusions:**

Whilst no PBMs comprehensively demonstrated evidence of construct validity and responsiveness in cataract surgery patients, the ICECAP-O was the most responsive generic PBM to improvements in QOL. Surprisingly the EQ-5D-5L was not more responsive than the EQ-5D-3L in this setting.

**Electronic supplementary material:**

The online version of this article (10.1007/s11136-020-02443-3) contains supplementary material, which is available to authorized users.

## Introduction

A cataract is an opacity of the eye lens that is the leading worldwide cause of blindness [1]. It can be successfully treated using surgery, which is the most common operation conducted in many countries [2]. Cataract surgery rates range from up to 10,000 operations per million population in 1 year in developed countries (e.g. USA) to less than 500 in developing countries (e.g. Ethiopia, Kenya) [2].

The National Institute for Health and Care Excellence (NICE) recommends the use of economic evaluation to inform decision-making about which treatments to fund [3]. For the evaluation of interventions funded by the NHS, cost–utility analysis (CUA) is the preferred method of economic evaluation and the quality-adjusted life year (QALY) is the preferred measure of benefit [3]. The ‘quality adjustment’ can be derived from preference-based measures (PBMs). Scores on these questionnaires are weighted to reflect the value the general population has placed on a particular state of health. PBMs can be used to value the effects of healthcare interventions in one index measure. PBMs could reflect generic health-related quality of life (HRQL) (e.g. EQ-5D), generic measures of HRQL expanded to include disease-specific dimensions (e.g. EQ-5D ‘bolt-ons’) or measures broader than HRQL such as capability wellbeing (e.g. ICECAP). This study sought to explore which PBMs are most appropriate in a cataract patient population.

The EQ-5D [4] is endorsed by NICE [3]. The first iteration of the measure, the EQ-5D-3L, comprises five ‘core’ questions about five domains of HRQL, each question has three response options. These domains are mobility, self-care, usual activities, pain and anxiety/depression. There is criticism however that the EQ-5D-3L is insensitive [5, 6] and in non-acute conditions, a significant number of patients score the highest value of one (ceiling effect) [6]. In response to these and other concerns, the EQ-5D-5L was developed. The EQ-5D-5L [7] increases the possible response options from three to five levels and modifies some wording. There are currently two algorithms to generate EQ-5D-5L preference-based utilities for a UK sample; A Value Set for England (EQ-5D-5L-VSE) [8] and the EQ-5D Crosswalk (EQ-5D-5L-CW) [9]). NICE recently confirmed their position that the EQ-5D-5L-CW should be used to generate utilities [10].

Another criticism is that the EQ-5D domains are not relevant to certain conditions, including visual impairment [11, 12]. Consequently, an EQ-5D-3L vision bolt-on was developed (EQ-5D-3L + VIS) [12], asking a sixth question about their vision (using glasses or contact lenses if needed). The item is worded as follows: I have no/some/extreme problems seeing. Methodological issues potentially impeding bolt-on implementation include small samples used to value the bolt-on, no validation of value sets and that they appear to impact responses to ‘core’ EQ-5D domains [12, 13].

The capability approach offers an alternative to HRQL, where an individual’s ability, or capability to function is the outcome evaluated [14]. Cataracts may limit individuals’ ability to live fulfilling lives, but successful cataract surgery could reduce limitations caused by impaired vision. Capability measures may better capture these benefits compared to HRQL outcomes. The ICECAP-O [15] measures this construct in older adults, but it has not been used in a cataract patient population [16]. The ICECAP-O’s five domains cover attachment (love and friendship), security (thinking about the future without concern), role (doing things that make you feel valued), enjoyment and control (independence). Each attribute has four response options. The ICECAP-O has been valued in the UK using a best–worst scaling approach [17], in contrast to the EQ-5D which used time trade off (TTO) (EQ-5D-3L, EQ-5D + 3L + VIS) and TTO/discrete choice methods (EQ-5D-5L).

Assessing the cost-effectiveness of cataract treatment options requires a PBM suitable for use in this population. Previous studies have assessed the responsiveness of PBMs in cataract patients undergoing surgery [18, 19], however, they had relatively small sample sizes (< 400) and none have included a measure of capability wellbeing. The questions of which PBM to use in cataract surgery patients and whether health is the most appropriate outcome, remain to be answered. Brazier et al. [20] recommend that a PBM is chosen based on the psychometric performance (including construct validity and responsiveness) in the patient population. Whilst the EQ-5D has been used in a cataract population before [18, 21], there is currently no published evidence of the use of the ICECAP-O [16]. The objective of this analysis was to evaluate the construct validity and responsiveness of the EQ-5D-3L, EQ-5D-5L, EQ-5D-3L + VIS and ICECAP-O in a cataract patient population.

## Methods

### Participants

The analyses used data from the Predict-CAT study, a cohort study of cataract surgery patients. Eligible participants were aged 50 or over, were able to understand and complete the PBMs, and were approaching either their first cataract surgery on either eye or a second surgery on the fellow eye. Participants were recruited from two NHS trusts (University Hospitals Bristol NHS Foundation Trust and Gloucestershire Hospitals NHS Foundation Trust) in the South West of England at the time of being listed for cataract surgery or at a pre-operative assessment appointment.

### Data collection

Participants attended two study visits, before and after surgery. The post-operative appointment was scheduled to take place 6–8 weeks after cataract surgery, although in practice there was some variation. All participants completed the Cat-PROM5 and ICECAP-O. The Cat-PROM5 is a five-item questionnaire designed to measure the HRQL impact of cataract surgery [22]. The Cat-PROM5 is responsive to changes in vision following cataract surgery (Cohen’s d = –1.45) [22], although is not preference based and thus cannot be used in CUA. It is currently being piloted in the National Ophthalmology Database Cataract Surgery Audit [23], its use having been encouraged by NICE [24]. Cat-PROM5 is comparable or performs better than the widely used CATQUEST-9SF nine item questionnaire [25].

For the remaining measures, participants were randomised in a 1:1:1 allocation to complete either the EQ-5D-3L, EQ-5D-3L + VIS or the EQ-5D-5L. Randomisation used an automated allocation when participants were added to the study database. Data collected also included socio-demographic information, medical history, assessment of visual function, and an ocular examination with pupil dilation.

A description of the measures and their scoring is provided in the Supplementary material. Lower Cat-PROM5 scores reflect better quality of life (QOL), whereas higher PBM scores are better. Both the EQ-5D-5L-CW [9] and EQ-5D-5L-VSE [8] algorithms were used to score the EQ-5D-5L.

### Descriptive statistics

Clinical (visual acuity, diabetic status, first or second eye surgery, complications) and socio-demographic characteristics (age, gender) of the sample were summarised. Descriptive statistics were generated for PBM indices at baseline and follow-up. We estimated the proportion of participants scoring the maximum and minimum scores at baseline. A threshold of 15% of was chosen [26] to define potentially problematic ceiling (maximum) or floor (minimum) effects. Large proportions of patients reporting the highest or lowest value at baseline reduces the potential to demonstrate either improvement or decline in condition following cataract surgery.

### Construct validity

#### Convergent validity

Convergent validity is the association between the measures of interest and outcomes measuring the same or overlapping constructs. Spearman’s rank correlations were calculated between all PBM and Cat-PROM5 scores at baseline. Correlation coefficients were interpreted using Cohen’s thresholds (large >  ± 0.5, ± 0.5–0.3 moderate, ± 0.3–0.1 small, <  ± 0.1 insubstantial) [27, 28]. The relationship between visual acuity, measured using a LogMAR chart, and the PBMs was explored by measuring Spearman’s correlations between the PBMs and habitual near visual acuity in the eye to be operated on (referred to as operated eye at baseline hereafter).

#### Known-groups validity

Known-groups compare the outcome measure in groups that are expected to differ. Three group comparisons were chosen based on previous research. These were (1) whether it was participants’ first or second eye surgery (baseline scores, second eye surgery participants expected to have higher HRQL/capability) [11], (2) visual acuity as good (≤ 0.3 LogMAR) or poor (> 0.3 LogMAR) monocular habitual near visual acuity (baseline scores, patients with poorer visual acuity expected to have worse HRQL/capability) [29, 30], and ocular comorbidities (baseline scores, participants with comorbidities expected to have worse HRQL/capability) [31]. Linear regressions were conducted to compare scores between known-groups. This approach is commonly used when analysing utility scores bounded at one [32, 33]. The group was the predictor and PBM scores the dependent variables. Covariates in all regressions were age, gender and diabetic status. Analyses were stratified by EQ-5D randomisation group; thus ICECAP-O known-group differences were tested three times. This eliminated the potential for the ICECAP-O to appear to perform better simply due to the larger sample completing that measure.

### Responsiveness

A PBM is responsive if changes in the index score reflect known changes in health [20]. These changes are defined using external indicators (anchors) of either clinical or patient-reported change, but they must be relevant to the condition. After surgery, patients completed two questions that asked about their perceived benefit of surgery and change in visual QOL. These were appended to the Cat-PROM5 post-operative questionnaire. These response options were used as anchors and participants were categorised into the following groups:

Perceived benefit of surgery

- I have gained significant benefit
- I have not gained significant benefit/I am worse off

Change in visual QOL

- Visual QOL has improved significantly
- Visual QOL has not changed by a significant amount/it’s worse

The no change/worsening response options were combined due to few participants reporting worsening [perceived benefit *N* = 34 (2.81%), visual QOL *N* = 27 (2.23%)].

Change in visual acuity was used as a clinical anchor. Based on a change in monocular habitual near visual acuity threshold (− 0.2 LogMAR), participants were categorised as either improving or experiencing no change/worsening. This was based on clinical expertise, previous literature [34] and data from the National Ophthalmology Database Audit [23].

For each PBM, change in PBM index was calculated between baseline and follow-up for each patient. Mean difference was compared between groups gaining significant benefit and those not changing by a significant amount/worse off using analysis of covariance (ANCOVA). Covariates included age, gender, diabetic status and complications.

Effect sizes were calculated for each PBM index score. These statistics quantify the difference between pre- and post-surgery scores in standardised units, enabling the comparison of the PBMs. Effect size is the change score divided by the standard deviation at baseline (Cohen’s d). Where no comparative data are available, effect sizes can be interpreted using Cohen’s thresholds [27, 28]. These are 0.20 small change, 0.50 moderate change and 0.80 large change [27]. Effect sizes for participants worsening/experiencing no benefit were expected to be less than those experiencing benefit. An effect size smaller than 0.2 would be expected when no change/worsening occurred.

### Evaluating the performance of the PBMs

We examined the construct validity and responsiveness of the PBMs using the properties reported in Brazier et al. [20].

The following criteria were tested.

- Less than 15% of participants would score the maximum or minimum score at baseline.
- At least moderate correlations (coefficients 0.3–0.5) were expected between generic PBMs and the Cat-PROM5 at baseline.
- PBMs would distinguish between the following known-groups at baseline: patients with good or poor vision, patients with and without ocular comorbidities and first or second eye surgery.
- Effect sizes of change would be less than 0.2 for participants experiencing no change or worsening in visual QOL or experiencing no benefit of surgery.
- Effect sizes of change would be greater than 0.2 for participants experiencing improvements in visual QOL and visual improvements.

## Results

3742 potentially eligible patients were approached to participate. Of these, 2230 (59.6%) declined and 6 (0.2%) were not eligible. Of the 1506 who consented, 191 (12.7%) did not complete baseline questionnaires. Table 1 presents baseline sample characteristics on the 1315 study participants. The characteristics appear to be balanced across randomisation groups, although slightly more participants completed the EQ-5D-5L at baseline than the other EQ-5D questionnaires. This was due to lower attrition between randomisation and baseline questionnaire completion in this group. In total, 105 (8%) patients did not provide any follow-up PBM data. The majority of participants were of White British ethnicity, did not have diabetes and were having their first cataract surgery. Approximately a quarter of participants had near visual acuity in their operated eye at baseline that could be described as ‘good’ (visual acuity ≤ 0.3 LogMAR).

**Table 1:** Baseline characteristics

	EQ-5D-3L + VIS	EQ-5D-3L	EQ-5D-5L	Whole sample
*N*	437	435	443	1315
Age, mean (SD)	73.3 (8.6)	74.2 (8.0)	74.2 (8.1)	73.9 (8.3)
Gender *N* (%)				
Male	223 (51.0%)	219 (50.3%)	214 (48.4%)	656 (49.9%)
Female	214 (49.0%)	216 (49.7%)	229 (51.6%)	659 (50.1%)
Previous cataract surgery *N* (%)				
No	269 (61.6%)	270 (62.1%)	288 (65.1%)	827 (62.9%)
Yes	168 (38.4%)	165 (37.9%)	155 (34.9%)	488 (37.1%)
Ethnicity *N* (%)				
White	403 (92.6%)	414 (95.2%)	427 (96.4%)	1244 (94.7%)
Non-White	32 (7.4%)	21 (4.8%)	16 (3.6%)	69 (5.3%)
Missing	2	0	0	2
Diabetes *N* (%)				
No	359 (82.2%)	344 (79.1%)	354 (80.0%)	1057 (80.4%)
Yes	78 (17.8%)	91 (20.9%)	89 (20.0%)	258 (19.6%)
Ocular comorbidities baseline *N* (%)				
No ocular Comorbidity/unknown	194 (44.4%)	194 (44.6%)	189 (42.6%)	577 (43.8%)
Ocular comorbidity	243 (55.6%)	241 (55.4%)	254 (57.4%)	738 (56.2%)
Baseline visual acuity (logMAR)*N* (%)				
≤ 0.3 (good)	99 (28.6%)	94 (26.7%)	100 (28.5%)	293 (27.9%)
> 0.3 (poor)	247 (71.4%)	258 (73.3%)	251 (71.5%)	756 (72.1%)
Missing	91	83	94	266
Baseline Cat-PROM5 Score mean (SD)	− 0.21 (2.38)	− 0.34 (2.36)	− 0.38 (2.21)	− 0.31 (2.32)

% are proportions of valid responses

Ocular comorbidities included are amblyopia, diabetic retinopathy, glaucoma, age-related macular degeneration, other macular pathology, other retinal vascular pathology, other

Visual acuity is monocular habitual near vision in the operated eye

All participants (i.e. the whole sample) completed the ICECAP-O and Cat-PROM5

### Descriptive statistics

Descriptive statistics for the PBMs are reported in Table 2. The EQ-5D-3L + VIS had the highest mean PBM index at both timepoints. All mean PBM scores increased between baseline and follow-up. Variability was greatest for the EQ-5D-3L, with the largest standard deviations observed for this measure. Variability for ICECAP-O and EQ-5D-3L + VIS were lowest.

**Table 2:** Descriptive statistics of PBM index scores at baseline and follow-up

	EQ-5D-3L + VIS		EQ-5D-3L		EQ-5D-5L-VSE		EQ-5D-5L-CW		ICECAP-O	
	Baseline	Follow-up	Baseline	Follow-up	Baseline	Follow-up	Baseline	Follow-up	Baseline	Follow-up
*N*^a^	436	405	435	403	439	400	439	400	1308	1207
Mean (SD)	0.87 (0.12)	0.91 (0.11)	0.76 (0.24)	0.80 (0.23)	0.82 (0.18)	0.85 (0.17)	0.76 (0.20)	0.79 (0.20)	0.86 (0.13)	0.89 (0.11)
Median	0.90	0.94	0.80	0.85	0.87	0.89	0.79	0.83	0.89	0.91
Minimum	0.28	0.32	− 0.18	− 0.08	− 0.20	− 0.15	− 0.24	− 0.29	0.16	0.16
Maximum	1	1	1	1	1	1	1	1	1	1
Ceiling *N* (%)	38 (8.72)		118 (27.13)		69 (15.72)		69 (15.72)		123 (9.40)	
Floor *N*	0		0		0		0		0	

Floor and ceiling effects were only calculated at baseline as it is only at this timepoint that a large effect would indicate potential inability of the PBM to measure improvement (ceiling) or deterioration (floor) in HRQL/capability wellbeing in this group of patients seeking healthcare for a vision problem

*PBMs* higher scores reflect better quality of life/capability, *EQ-5D-5L-VSE* EQ-5D-5L Value Set for England scoring algorithm, *EQ-5D-5L-CW* EQ-5D-5L Crosswalk scoring algorithm

^a^*N* = 5 participants completed ICECAP-O at baseline but EQ-5D data is missing

### Floor and ceiling effects

For the EQ-5D-3L, 27.1% (118/435) of participants scored one at baseline (index profile 11111), greatly exceeding the threshold indicating a potentially problematic ceiling effect. The EQ-5D-5L marginally exceeded the 15% threshold also (69/439, 15.7%). Of the generic PBMs, the ICECAP-O had the smallest ceiling effect (123/1308, 9.4%). The EQ-5D-3L + VIS was marginally lower (38/436, 8.7%). A high proportion of participants scored 0.961 on the EQ-5D-3L + VIS (74/436, 17.0%). This corresponds to an index profile of 111112 (‘no problems’ on all EQ-5D-3L domains and ‘some problems’ on the vision bolt-on domain). As usually observed, all PBM distributions were negatively skewed, with more participants reporting good health. No participants scored the lowest possible PBM score.

### Convergent validity

Correlation coefficients between the PBMs were all strong (> 0.5; Table 3). Moderate associations were observed between the Cat-PROM5 and the EQ-5D-3L + VIS, EQ-5D-5L-VSE and the ICECAP-O but not the EQ-5D-3L and EQ-5D-3L-CW. As expected, the correlation coefficient between the vision-specific EQ-5D-3L + VIS and Cat-PROM5 was largest. In regard to correlations between PBMs and visual acuity, the relationships were either small (0.1–0.3 for the EQ-5D-3L + VIS, EQ-5D-5L-VSE and EQ-5D-3L-CW) or insubstantial (< 0.1 for the ICECAP-O and EQ-5D-3L).

**Table 3:** Spearman’s correlation coefficients between PBMs, Cat-PROM5 and visual acuity (baseline data)

	EQ-5D-3L + VIS	EQ-5D-3L	EQ-5D-5L-VSE	EQ-5D-5L-CW	ICECAP-O	Cat-PROM5	Visual acuity
EQ-5D-3L + VIS	1						
EQ-5D-3L	N/A	1					
EQ-5D-5L-VSE	N/A	N/A	1				
EQ-5D-5L-CW	N/A	N/A	0.98	1			
ICECAP-O	0.50**	0.53**	0.56**	0.52**	1		
Cat-PROM5	− 0.42**	− 0.23**	− 0.31**	− 0.29**	− 0.35**	1	
Visual acuity	− 0.10	− 0.02	− 0.12*	− 0.11*	− 0.06*	0.14**	1

Visual acuity is monocular habitual near vision in the operated eye (LogMAR score)

*N/A* correlations not possible as participants completed only 1 version of the EQ-5D, *Cat-PROM5 and visual acuity* lower scores are better

*Correlation significant at 0.05 level

**Correlation significant at the 0.01 level

### Known-groups

For the previous cataract surgery and baseline visual acuity known-groups, the mean differences in PBM scores were small, but in the expected direction (Table 4). For the ocular comorbidities known-group, the mean differences in PBM scores were also small, and not consistently in the expected direction. In almost all analyses the confidence interval spanned zero.

**Table 4:** Linear regression analyses of known-groups validity

Randomisation group	PBM	Previous cataract surgery			*p* value
		No Mean (SD) *N*	Yes Mean (SD) *N*	MD (95% CIs)	
EQ-5D-3L + VIS	EQ-5D-3L + VIS	0.87 (0.13) 269	0.87 (0.12) 167	0.002 (− 0.022:0.026)	0.866
	ICECAP-O	0.85 (0.14) 268	0.87 (0.11) 166	0.018 (− 0.006:0.043)	0.141
EQ-5D-3L	EQ-5D-3L	0.75 (0.26) 270	0.79 (0.20) 165	0.038 (− 0.008:0.084)	0.103
	ICECAP-O	0.84 (0.14) 268	0.87 (0.12) 162	0.023 (− 0.003:0.049)	0.079
EQ-5D-5L	EQ-5D-5L-VSE	0.81 (0.20) 286	0.85 (0.14) 153	0.034 (− 0.002:0.069)	0.066
	EQ-5D-5L-CW	0.74 (0.21) 286	0.78 (0.17) 153	0.038 (− 0.002:0.077)	0.061
	ICECAP-O	0.86 (0.11) 286	0.88 (0.12) 153	0.020 (− 0.003:0.043)	0.082

Randomisation group		Visual acuity			*p* value
		Good (≤ 0.3 LogMAR) Mean (SD) *N*	Poorer (> 0.3 LogMAR) Mean (SD) *N*	MD (95% CIs)	
EQ-5D-3L + VIS	EQ-5D-3L + VIS	0.89 (0.10) 99	0.86 (0.13) 246	0.031 (0.002:0.061)	0.038
	ICECAP-O	0.88 (0.11) 99	0.86 (0.13) 246	0.022 (− 0.008:0.052)	0.146
EQ-5D-3L	EQ-5D-3L	0.78 (0.21) 94	0.75 (0.25) 258	0.037 (− 0.020:0.094)	0.205
	ICECAP-O	0.88 (0.09) 93	0.84 (0.14) 256	0.031 (0.000:0.063)	0.053
EQ-5D-5L	EQ-5D-5L-VSE	0.84 (0.19) 98	0.83 (0.17) 249	0.016 (− 0.025:0.056)	0.442
	EQ-5D-5L-CW	0.79 (0.21) 98	0.75 (0.19) 249	0.028 (− 0.017:0.073)	0.221
	ICECAP-O	0.88 (0.09) 98	0.86 (0.11) 249	0.010 (− 0.014:0.035)	0.407

Randomisation group		Ocular comorbidities			*p* value
		No Mean (SD) *N*	Yes Mean (SD) *N*	MD (95% CIs)	
EQ-5D-3L + VIS	EQ-5D-3L + VIS	0.86 (0.13) 193	0.87 (0.12) 243	0.008 (− 0.016:0.031)	0.518
	ICECAP-O	0.86 (0.12) 192	0.86 (0.13) 242	− 0.003 (− 0.027:0.021)	0.796
EQ-5D-3L	EQ-5D-3L	0.76 (0.25) 194	0.76 (0.23) 241	0.004 (− 0.041:0.049)	0.864
	ICECAP-O	0.86 (0.12) 190	0.84 (0.14) 240	− 0.015 (− 0.041:0.010)	0.230
EQ-5D-5L	EQ-5D-5L-VSE	0.84 (0.16) 185	0.81 (0.20) 254	− 0.023 (− 0.057:0.012)	0.193
	EQ-5D-5L-CW	0.77 (0.19) 185	0.75 (0.21) 254	− 0.018 (− 0.055:0.020)	0.356
	ICECAP-O	0.87 (0.11) 185	0.86 (0.12) 254	0.000 (− 0.022:0.021)	0.990

Visual acuity is habitual near vision in the operated eye (LogMAR score)

*MD* mean difference, adjusted for age, gender, diabetic status

### Responsiveness

For the improvement in QOL anchor, the mean difference in scores was in the expected direction for all PBMs, but the EQ-5D-5L mean difference was closer to zero and the confidence interval for that PBM included zero (Table 5). All PBMs identified moderate (EQ-5D-3L + VIS) or small (other PBMs) effect sizes in patients who reported QOL improvements. Unexpectedly, there was a small positive effect size for the EQ-5D-3L + VIS in patients who stated that their QOL had not improved.

**Table 5:** Responsiveness: comparisons of PBM and Cat-PROM5 change scores between anchors of change

Randomisation group	PBM	Mean change (SD) N	Effect size	Mean change (SD) *N*	Effect size	MD (95% CI)	*p*
	Visual QOL (post-operative questionnaire)						
		QOL not changed/worsened		QOL improved significantly			
EQ-5D-3L + VIS	EQ-5D-3L + VIS	0.030 (0.08) 163	0.25	0.054 (0.09) 239	0.41	0.023 (0.006: 0.040)	0.009
	ICECAP-O	0.006 (0.09) 162	0.05	0.039 (0.11) 239	0.28	0.035 (0.014: 0.055)	0.001
EQ-5D-3L	EQ-5D-3L	0.015 (0.20) 157	0.07	0.052 (0.20) 246	0.20	0.038 (− 0.004: 0.079)	0.075
	ICECAP-O	0.009 (0.10) 156	0.07	0.046 (0.11) 241	0.34	0.034 (0.012: 0.055)	0.002
EQ-5D-5L	EQ-5D-5L VSE	0.012 (0.11) 163	0.07	0.027 (0.13) 232	0.15	0.015 (− 0.010: 0.039)	0.242
	EQ-5D-5L CW	0.026 (0.13) 163	0.15	0.031 (0.15) 232	0.15	0.004 (− 0.025: 0.033)	0.796
	ICECAP-O	0.007 (0.09) 163	0.07	0.039 (0.09) 231	0.34	0.028 (0.010: 0.046)	0.003

Randomisation group	Perceived benefit of surgery (post-operative questionnaire)						
		No significant benefit/worsening		Gained significant benefit			
EQ-5D-3L + VIS	EQ-5D-3L + VIS	0.036 (0.08) 57	0.25	0.045 (0.08) 347	0.37	0.011 (− 0.013: 0.035)	0.373
	ICECAP-O	− 0.014 (0.11) 57	− 0.12	0.032 (0.10) 346	0.25	0.048 (0.020: 0.077)	0.001
EQ-5D-3L	EQ-5D-3L	0.014 (0.22) 54	0.06	0.042 (0.20) 349	0.17	0.025 (− 0.034: 0.084)	0.407
	ICECAP-O	0.008 (0.09) 54	0.05	0.035 (0.11) 343	0.27	0.022 (− 0.009: 0.053)	0.163
EQ-5D-5L	EQ-5D-5L VSE	0.013 (0.15) 59	0.07	0.022 (0.12) 336	0.13	0.007 (− 0.028: 0.041)	0.702
	EQ-5D-5L CW	0.026 (0.18) 59	0.13	0.030 (0.14) 336	0.16	0.001 (− 0.039: 0.042)	0.951
	ICECAP-O	− 0.017 (0.10) 59	− 0.16	0.033 (0.09) 335	0.30	0.048 (0.022: 0.073)	< 0.001

Randomisation group	Change in visual acuity (LogMAR change in habitual near visual acuity in operated eye)						
		Worsen/no change		Improved			
EQ-5D-3L + VIS	EQ-5D-3L + VIS	0.034 (0.07) 142	0.27	0.043 (0.08) 198	0.34	0.008 (− 0.009: 0.025)	0.377
	ICECAP-O	0.022 (0.09) 142	0.18	0.025 (0.09) 198	0.20	0.004 (− 0.017: 0.024)	0.714
EQ-5D-3L	EQ-5D-3L	0.026 (0.22) 128	0.11	0.042 (0.20) 208	0.18	0.009 (− 0.038: 0.056)	0.695
	ICECAP-O	0.026 (0.10) 127	0.21	0.036 (0.12) 205	0.27	− 0.001 (− 0.026: 0.024)	0.909
EQ-5D-5L	EQ-5D-5L VSE	0.026 (0.13) 134	0.14	0.020 (0.12) 205	0.13	− 0.007 (− 0.034: 0.021)	0.69
	EQ-5D-5L CW	0.036 (0.15) 134	0.16	0.026 (0.14) 205	0.15	− 0.013 (− 0.045: 0.020)	0.478
	ICECAP-O	0.018 (0.08) 133	0.17	0.034 (0.09) 205	0.30	0.015 (− 0.005: 0.034)	0.134

*MD* mean difference, adjusted for age, gender, diabetic status and complications

For the perceived benefit of surgery anchor, the mean difference in scores was again in the expected direction for all PBMs. However, the mean difference was largest for the ICECAP-O and that was the only PBM where the confidence interval excluded zero. All PBMs identified small effect sizes in patients who reported significant benefit from surgery. Again, there was a small positive effect size for the EQ-5D-3L + VIS in patients who stated that they had no significant benefit from surgery.

For the visual acuity anchor, mean differences in PBM scores were close to zero. The EQ-5D-3L + VIS and ICECAP-O identified small-positive effect sizes in patients whose visual acuity improved. For patients with little or no improvement in visual acuity, effect sizes were similar across all PBMs.

### Summary

The EQ-5D-3L and EQ-5D-5L did not perform well across almost every measure of validity and responsiveness and had the largest ceiling effects (Table 6). The EQ-5D-3L + VIS had a lower ceiling effect and better convergent validity with the Cat-PROM5. It was able to differentiate between patient groups who did and did not report benefit from surgery and improved visual QOL after surgery. However, it also identified small positive effect sizes in patients who reported no benefit or no improved visual QOL after surgery. The ICECAP-O also had a low ceiling effect and there was some evidence of convergent validity with the Cat-PROM5. It performed best on many measures of responsiveness.

**Table 6:** Summary of PBM performance against criteria evaluated

Criteria	PBMs				
	EQ-5D-3L + VIS	EQ-5D-3L	EQ-5D-5L VSE	EQ-5D-5L-CW	ICECAP-O
Ceiling effect	✓	✗	✗	✗	✓
Floor effect	✓	✓	✓	✓	✓
Convergent validity					
Cat-PROM5 correlation	✓	✗	✓	✗	?^a^
Visual acuity correlation	✗	✗	✗	✗	✗
Known-groups validity					
First eye or second eye surgery	✗	✗	✗	✗	✗
Habitual near visual acuity in the operated eye (logMAR)	✓	✗	✗	✗	?^b^
Ocular comorbidities	✗	✗	✗	✗	✗
Responsiveness					
Change scores and effect sizes					
Visual QOL	✗	✗	✗	✗	✓
Patient perceived benefit of surgery	✗	✗	✗	✗	?^c^
Change in near visual acuity in operated eye	✗	✗	✗	✗	✗

Visual QOL and Patient perceived benefit obtained from post-operative supplementary questionnaire

Ceiling effect—greater than 15% scoring the maximum of one

First eye or second eye surgery—Second eye surgery patients were expected to report significantly better HRQL

Habitual near visual acuity in the operated eye (logMAR)—Patients with worse visual acuity were expected to report significantly lower HRQL

Ocular comorbidities—Patients with ocular comorbidities were expected to report lower HRQL

Floor effect—greater than 15% scoring the minimum possible score

? indicates conflicting results for EQ-5D randomisation groups

^a^EQ-5D-3L group correlation coefficient did not exceed 0.3

^b^Between group differences significant (*p* < 0.05) for the EQ-5D-3L group only

^c^No difference in change scores for the EQ-5D-3L group

## Discussion

### Principal findings

Predict-CAT is a large cohort study that resulted in a detailed dataset describing the patient-reported impact of cataracts before and after surgery. The core EQ-5D measures did not perform well across the tests of validity and responsiveness conducted. There was little evidence that the EQ-5D-5L is more responsive than the EQ-5D-3L. The ICECAP-O was more responsive than the EQ-5D measures to post-operative improvements in visual QOL and the perceived benefit of surgery, although the effect sizes were small. None of the PBMs were responsive to changes in visual acuity.

### Strengths and weaknesses

This is the first published use of the ICECAP-O in cataract patients and the first that allows the comparison of the EQ-5D-5L, EQ-5D-3L and the EQ-5D-3L + VIS. This large cohort was mostly representative of UK cataract surgery patients, with a similar median age (75) and baseline visual acuity (0.5 LogMAR) as the UK National Ophthalmology Database Audit 2018 [23] (median age 76.3, visual acuity 0.5 LogMAR). The audit data comprised 50% of UK cataract surgeries undertaken in 2017–2018. Whilst the EQ-5D-3L + VIS has not been used extensively, there is ongoing interest in the development of EQ-5D bolt-ons to fill perceived gaps in the core measures [35]. There is also considerable debate about which EQ-5D version and scoring algorithm should be used to measure self-reported health [36] and to inform decision-making [10]. Another strength is the patient-reported and objective measures of change in visual acuity collected in the study. It could be argued that patient perceived benefits are the outcome that should be targeted, as these might not correspond directly to clinical change. This study was able to test the responsiveness of the PBMs to both of these outcomes, replicating findings that visual acuity is not associated with generic PBMs [37–39].

A limitation of the study is that the three versions of the EQ-5D questionnaire were completed by different patient cohorts. If participants were to have completed every questionnaire, response burden would have been excessive. These cohorts were randomly assigned, relatively large and had similar baseline characteristics, nevertheless it is possible that some observed differences in validity and responsiveness might be due to chance. In addition, the study comprised cataract patients only. All participants had surgery, so experienced some change in clinical condition. Including a control group of cataract patients on the waiting list for surgery might have provided a more robust assessment of responsiveness. When evaluating the PBM performance, judgements were made on a series of thresholds and statistical tests. In some cases, decisions were based on marginal results. Whilst using arbitrary cut-offs is perhaps crude, decisions made on the triangulation of evidence is also subjective [40].

### Comparison with existing research

The EQ-5D-3L ceiling effect at baseline was larger than previous studies in cataract patients 19.3% [41] 23% [42]), although not as pronounced as that observed by Gandhi et al. [18] (51%). The EQ-5D-5L ceiling effect reported by Gandhi et al. [18] (46%) also exceed the Predict-CAT results. Gandhi et al. [18] reported the performance of the EQ-5D-5L and EQ-5D-3L in a small cohort (*n* = 148) of cataract surgery patients and similarly found the EQ-5D measures to be inferior to alternative PBMs (HUI3 and SF-6D). The EQ-5D-5L was scored using four algorithms. Gandhi et al. [18] concluded the EQ-5D-5L is the preferred version due to its superior responsiveness (irrespective of scoring algorithm), however, our study does not support this. Gandhi et al. [18] did not examine responsiveness in relation to change in either patient-reported or objectively measured vision. Comparing the EQ-5D-5L scoring algorithms, EQ-5D-5L-VSE utilities were greater than the EQ-5D-5L-CW obtained values in our study. This is consistent with published comparisons [43]. Despite the addition of two more response categories, the five-level version showed no advantage over the three level. Neither version was consistently better when examining responsiveness, which is compatible with the mixed evidence available thus far [36].

### Meaning of the study

This is the first study to administer the EQ-5D-3L, EQ-5D-3L + VIS, EQ-5D-5L and ICECAP-O concurrently and in a longitudinal study. Furthermore, the two available EQ-5D-5L scoring algorithms were applied [8, 9]. The addition of the vision bolt-on appears to improve the responsiveness of the EQ-5D-3L in this patient population, however it also seems responsive to the process of surgery in the absence of benefit. It was also the only EQ-5D variant to discriminate between participants with poorer and good visual acuity. The anchors used to measure patient-reported change required a reflection on their condition pre-surgery. This may introduce recall bias. In addition, assessing responsiveness as the difference between two assessments of your ‘health today’ perhaps does not reflect the change attributable to surgery. Complications or other negative experiences might be missed for example.

The poor association between vision and HRQL highlights challenges interpreting and appraising evidence of construct validity and responsiveness of PBMs. Firstly, PBMs have been valued by the general population, but clinical outcomes and other patient-reported outcomes are not. Associations between these measures might be improbable as a result. Furthermore, PBMs measure aspects of health and wellbeing unrelated to the condition. They are therefore not intended to be strongly associated with condition specific measures or clinical outcomes. Irrespective of this, there are certain properties that we would expect of a PBM. These include a small ceiling effect among a group of patients seeking care for visual problems affecting their QOL and being able to differentiate between patients who do and do not report improved QOL after a procedure of proven effectiveness, like cataract removal. The problems are probably related, with a high ceiling effects for the EQ-5D-5L and EQ-5D-3L leading to a lack of responsiveness.

### Unanswered questions and future research

It seems that the EQ-5D-3L or EQ-5D-5L should not be the sole PBMs used in studies evaluating the cost-effectiveness of cataract surgery. This analysis has revealed evidence of limited responsiveness and poor construct validity in both EQ-5D-3L and EQ-5D-5L amongst cataract patients. This should be reflected in interpretations of cost-effectiveness analysis of interventions in cataract surgery patients. There is currently no evidence of the ICECAP-O’s content validity in this patient group. This was not within the remit of this study, but future qualitative work could be conducted to explore this. Future work could explore the suitability and performance of the ICECAP-A [44] in cataract patients given the potential importance of capability wellbeing in this context. The ICECAP-A measures capability wellbeing in adults as opposed to the focus on older adults in the ICECAP-O. Developed using qualitative research with UK adults, the five domains cover similar capabilities to the ICECAP-O, with some items reworded or the focus changed.

Whilst the ICECAP-O seems to be more responsive than the EQ-5D in cataract surgery patients, it cannot be used to generate QALYs. Yet without further methodological developments, neither can the EQ-5D-3L + VIS. There is no five-level vision bolt-on available, meaning a revised bolt-on is required, with concurrent robust valuation and validation. The methodological rigour and resources required to develop and value a bolt-on item challenges the feasibility of developing one for every condition that the EQ-5D is reportedly unsuitable. The endorsement of the EQ-5D for use in economic evaluation is largely justified by the need for a comparable measure of benefit. Bolt-ons lack comparability with core EQ-5D scores, however. Developing measures that are sufficiently broad to measure health-related wellbeing in all common conditions, without the need for bolt-ons should be prioritised. Finally, the ability to conclude what the best PBM is in cataract surgery patients should be informed by evidence comparing all PBMs available, such as the SF-6D [45] and HUI [46].

## Conclusion

The Predict-CAT study intended to identify a suitable PBM for use in patients undergoing cataracts surgery. Referring to the psychometric properties suggested by Brazier et al. [20] for selecting PBMs cost-effectiveness models, no PBMs showed convincing evidence of all properties. While the ICECAP-O appears to be the most responsive generic PBM to improvements in QOL following cataract surgery, evidence of known-groups validity was consistently poor in all PBMs. There was no evidence that the EQ-5D-5L was more responsive than the EQ-5D-3L in cataract surgery patients, despite the increased number of response categories. This study suggests that the generic EQ-5D-3L and EQ-5D-5L may not reflect the patient benefits of cataract surgery when used in CUA. Where data allows, additional analyses using broader outcomes (e.g. ICECAP-O or EQ-5D-3L + VIS) should be presented to enable informed decision-making where CUA using EQ-5D data is recommended.

## Electronic supplementary material

Below is the link to the electronic supplementary material.

Supplementary file1 (PDF 73 kb)

## References

[1] Flaxman SR, Bourne RRA, Resnikoff S, Ackland P, Braithwaite T, Cicinelli MV (2017). Global causes of blindness and distance vision impairment 1990–2020: A systematic review and meta-analysis. The Lancet Global Health.

[2] Wang W, Yan W, Fotis K, Prasad NM, Lansingh VC, Taylor HR (2017). Cataract surgical rate and socioeconomics: A global studycataract surgical rate and socioeconomics. Investigative ophthalmology & visual science.

[3] NICE (2013). A guide to the methods of technology appraisal.

[4] Brooks R (1996). EuroQol: The current state of play. Health Policy.

[5] Devlin NJ, Brooks R (2017). EQ-5D and the EuroQol Group: Past, present and future. Applied Health Economics and Health Policy.

[6] Payakachat N, Ali MM, Tilford JM (2015). Can the EQ-5D detect meaningful change? A systematic review. PharmacoEconomics.

[7] Herdman M, Gudex C, Lloyd A, Janssen MF, Kind P, Parkin D (2011). Development and preliminary testing of the new five-level version of EQ-5D (EQ-5D-5L). Quality of Life Research.

[8] Devlin NJ, Shah KK, Feng Y, Mulhern B, van Hout B (2018). Valuing health-related quality of life: An EQ-5D-5L value set for England. Health Economics.

[9] van Hout B, Janssen MF, Feng Y-S, Kohlmann T, Busschbach J, Golicki D (2012). Interim scoring for the EQ-5D-5L: Mapping the EQ-5D-5L to EQ-5D-3L value sets. Value in Health.

[10] NICE. (2019). Position statement on use of the EQ-5D-5L valuation set for England (updated October 2019).

[11] Fung SSM, Luis J, Hussain B, Bunce C, Hingorani M, Hancox J (2016). Patient-reported outcome measuring tools in cataract surgery: Clinical comparison at a tertiary hospital. Journal of Cataract & Refractive Surgery.

[12] Longworth L, Yang Y, Young T, Mulhern B, Hernandez Alava M, Mukuria C (2014). Use of generic and condition-specific measures of health-related quality of life in NICE decision-making: A systematic review, statistical modelling and survey. Health Technology Assessment.

[13] Yang Y, Rowen D, Brazier J, Tsuchiya A, Young T, Longworth L (2015). An exploratory study to test the impact on three “bolt-on” items to the EQ-5D. Value in Health.

[14] Coast J, Smith R, Lorgelly P (2008). Should the capability approach be applied in Health Economics?. Health Economics.

[15] Grewal I, Lewis J, Flynn T, Brown J, Bond J, Coast J (2006). Developing attributes for a generic quality of life measure for older people: Preferences or capabilities?. Social Science & Medicine.

[16] Proud L, McLoughlin C, Kinghorn P (2019). ICECAP-O, the current state of play: A systematic review of studies reporting the psychometric properties and use of the instrument over the decade since its publication. Quality of Life Research.

[17] Coast J, Flynn TN, Natarajan L, Sproston K, Lewis J, Louviere JJ (2008). Valuing the ICECAP capability index for older people. Social Science & Medicine.

[18] Gandhi M, Ang M, Teo K, Wong CW, Wei YC-H, Tan RL-Y (2019). EQ-5D-5L is more responsive than EQ-5D-3L to treatment benefit of cataract surgery. The Patient - Patient-Centered Outcomes Research.

[19] Kaplan RM, Tally S, Hays RD, Feeny D, Ganiats TG, Palta M (2011). Five preference-based indexes in cataract and heart failure patients were not equally responsive to change. Journal of Clinical Epidemiology.

[20] Brazier J, Ara R, Rowen D, Chevrou-Severac H (2017). A review of generic preference-based measures for use in cost-effectiveness models. PharmacoEconomics.

[21] Ang M, Fenwick E, Wong TY, Lamoureux E, Luo N (2013). Utility of EQ-5D to assess patients undergoing cataract surgery. Optometry and Vision Science.

[22] Sparrow J, Grzeda M, Frost N, Johnston R, Liu C, Edwards L (2018). Cat-PROM5: A brief psychometrically robust self-report questionnaire instrument for cataract surgery. Eye.

[23] The Royal College of Ophthalmologists National Ophthalmology Database Cataract Surgery Audit. Retrieved June 4, 2019 from https://www.nodaudit.org.uk/.

[24] NICE (2019). Quality statement 2: Referral for cataract surgery.

[25] Sparrow JM, Grzeda MT, Frost NA, Johnston RL, Liu CSC, Edwards L (2018). Cataract surgery patient-reported outcome measures: A head-to-head comparison of the psychometric performance and patient acceptability of the Cat-PROM5 and Catquest-9SF self-report questionnaires. Eye.

[26] Nunnally JC, Bernstein IH, Berge JMT (1967). Psychometric theory.

[27] Cohen J (1988). Statistical power analysis for the behavioral sciences.

[28] Thompson B (2007). Effect sizes, confidence intervals, and confidence intervals for effect sizes. Psychology in the Schools.

[29] Kobelt G, Lundstrom M, Stenevi U (2002). Cost-effectiveness of cataract surgery. Method to assess cost-effectiveness using registry data. Journal of Cataract and Refractive Surgery.

[30] Lundström M, Pesudovs K (2009). Catquest-9SF patient outcomes questionnaire: Nine-item short-form Rasch-scaled revision of the Catquest questionnaire. Journal of Cataract & Refractive Surgery.

[31] Grimfors M, Mollazadegan K, Lundström M, Kugelberg M (2014). Ocular comorbidity and self-assessed visual function after cataract surgery. Journal of Cataract & Refractive Surgery.

[32] Hernández M, Wailoo AJ, Ara R (2012). Tails from the peak district: Adjusted Limited Dependent Variable Mixture Models of EQ-5D Questionnaire health state utility values. Value in Health.

[33] Pullenayegum EM, Tarride J-E, Xie F, Goeree R, Gerstein HC, O'Reilly D (2010). Analysis of health utility data when some subjects attain the upper bound of 1: Are tobit and CLAD models appropriate?. Value in Health.

[34] Rosser DA, Cousens SN, Murdoch IE, Fitzke FW, Laidlaw DAH (2003). How sensitive to clinical change are ETDRS logMAR visual acuity measurements?. Investigative Ophthalmology & Visual Science.

[35] Finch AP, Brazier JE, Mukuria C (2019). Selecting bolt-on dimensions for the EQ-5D: Examining their contribution to health-related quality of life. Value in Health.

[36] Buchholz I, Janssen MF, Kohlmann T, Feng Y-SJP (2018). A systematic review of studies comparing the measurement properties of the three-level and five-level versions of the EQ-5D. Pharmacoeconomics.

[37] Datta S, Foss AJE, Grainge MJ, Gregson RM, Zaman A, Masud T (2008). The importance of acuity, stereopsis, and contrast sensitivity for health-related quality of life in elderly women with cataracts. Investigative Ophthalmology & Visual Science.

[38] Polack S, Eusebio C, Fletcher A, Foster A, Kuper H (2010). Visual impairment from cataract and health related quality of life: Results from a case-control study in the Philippines. Ophthalmic Epidemiology.

[39] Polack S, Kuper H, Mathenge W, Fletcher A, Foster A (2007). Cataract visual impairment and quality of life in a Kenyan population. British Journal of Ophthalmology.

[40] Ioannidis JPA (2019). The importance of predefined rules and prespecified statistical analyses: Do not abandon significance. JAMA.

[41] Ferreira LN, Ferreira PL, Pereira LN (2014). Comparing the performance of the SF-6D and the EQ-5D in different patient groups. Acta Medica Portuguesa.

[42] Allepuz A, Espallargues M, Moharra M, Comas M, Pons JMV, Research Group on Support Instruments, I. N. (2008). Prioritisation of patients on waiting lists for hip and knee arthroplasties and cataract surgery: Instruments validation. BMC Health Services Research.

[43] Mulhern B, Feng Y, Shah K, Janssen MF, Herdman M, van Hout B (2018). Comparing the UK EQ-5D-3L and English EQ-5D-5L value sets. PharmacoEconomics.

[44] Al-Janabi H, Flynn NT, Coast J (2012). Development of a self-report measure of capability wellbeing for adults: The ICECAP-A. Quality of Life Research.

[45] Brazier JE, Roberts J (2004). The estimation of a preference-based measure of health from the SF-12. Medical Care.

[46] Horsman J, Furlong W, Feeny D, Torrance G (2003). The Health Utilities Index (HUI): Concepts, measurement properties and applications. Health and Quality of Life Outcomes.

