# Supplementary material for: Assessing the construct validity and responsiveness of Preference-Based Measures (PBMs) in cataract surgery patients
Source: Qual Life Res. 2020 Feb 20;29(7):1935–46. doi: 10.1007/s11136-020-02443-3 (PMC7295830; doi:10.1007/s11136-020-02443-3)
Supplement: Supplementary file 1 — Supplementary file1 (PDF 73 kb) [file 11136_2020_2443_MOESM1_ESM.pdf]

K Breheny, W Hollingworth, R Kandiyali, P Dixon, A Loose, P Craggs, J Sparrow. Assessing the construct validity and responsiveness of Preference Based Measures (PBMs) in cataract surgery patients. Quality of Life Research.

Corresponding author: Katie Breheny, University of Bristol. katie.breheny@bristol.ac.uk

Supplementary material

*PBM and HRQL Questionnaire Scoring and Development*

| PBM | Construct measured | Target population | Description | Scoring | Used in economic evaluation | Use in cataracts patients | Minimum possible score | Maximum possible score | Recall period | Valuation method |
|---|---|---|---|---|---|---|---|---|---|---|
| EQ-5D-3L (4) | HRQL | Generic | Five-dimension PBM measuring health status. Questions have 3 response options and address domains of usual activities, mobility, anxiety/depression, self-care, pain/discomfort. | PBM | Yes | Yes | -0.594 | 1 | Today | TTO |
| EQ-5D-3L+VIS (12) | HRQL | Visual problems | Six-dimension PBM measuring health status and visual difficulties. Developed in response to criticism that the EQ-5D was insensitive to visual | PBM | No | Yes (validation sample) | 0.284 | 1 | Today | TTO |

| | | | | | | | | | | |
|---|---|---|---|---|---|---|---|---|---|---|
| | | | problems. It comprises the EQ-5D-3L, and an additional vision-specific question. | | | | | | | |
| EQ-5D-5L CW (9) EQ-5D-5L VSE (8) | HRQL | Generic | Updated version of the EQ-5D-3L. Response options were increased from 3 to 5. Two scoring algorithms are available. 1. A Value Set for England 2. EQ-5D-3L crosswalk. | PBM | Yes | Yes | -0.594 CW -0.285 VSE | 1 | Today | TTO (CW) TTO and DCE (VSE) |
| ICECAP-O (17) | Capability wellbeing | Older people aged 65+ | A PBM for use in older adults, measuring broader wellbeing beyond health. It has five attributes cover attachment, security, role, enjoyment and control. Each attribute has 4 response options. | PBM | Yes | No | 0 | 1 | At the moment | BWS |
| Cat-PROM5 (22) | Visual difficulties | Cataracts | A five item, Rasch-scored cataract-specific patient | Rasch scored | No | Yes | -9.18 | 7.45 | One month | N/A |

| | | |
|---|---|---|
| | reported outcome designed to measure visual difficulty from cataracts and relief from surgery. Response options range from 4 to 7. | |

PBM – Preference based measure

TTO – Time trade off

DCE- Discrete choice experiment

BWS – Best-worst scaling

CW – EQ-5D-5L Crosswalk scoring algorithm

VSE – EQ-5D-5L Value set for England scoring algorithm
